# Optimising Patient Safety During the Use of Intraoperative Imaging for the Surgical Management of Facial Fractures: A Pilot Study

**DOI:** 10.3390/cmtr19020026

**Published:** 2026-05-25

**Authors:** Nicole Garcia, Mohamed Badawy, Jake DiPasquale, Simon Maciburko, Marc Seifman

**Affiliations:** 1 Department of Plastic and Reconstructive Surgery, Eastern Health, Melbourne, VIC 3128, Australia; 2 Monash Health Imaging, Monash Health, Melbourne, VIC 3168, Australia; 3 Department of Medical Imaging and Radiation Sciences, Monash University, Melbourne, VIC 3800, Australia; 4 Department of Medical Imaging, Eastern Health, Melbourne, VIC 3128, Australia; 5 Department of Surgery, Monash University, Melbourne, VIC 3128, Australia

**Keywords:** facial fracture, computed tomography, radiation, intra-operative

## Abstract

The use of intraoperative computerised tomography (CT) to aid surgical management of facial fractures obviates the need for postoperative surgical scans, decreases return to theatre rates, and subsequently decreases overall hospital stay. This allows for better appreciation of a complex operative landscape in almost real time, and may become the gold standard of treatment. As with radiological investigations, decreasing radiation exposure of patients is a key goal. The aim of this study was to examine the effective radiation doses in patients undergoing surgical management of their facial fractures with the aid of a fixed-arm CT. This retrospective study was conducted on patients who underwent surgical fixation of their facial fractures within a hybrid surgical suite equipped with a fixed-arm cone beam CT (CBCT) from July 2023 to November 2024. The CBCT was used to assess adequacy of bony fixation. Data from imaging was collected to assess total effective radiation dose. Data from 30 random patients who underwent standard CT facial bones (CTFB) were collected as control. Data from 24 patients was collected. The majority were male (17/24, 70.8%) with an age range of 20–94 years. The average dose of the effective doses calculated in the CTFB cohort was 0.64 mSv (SD 0.05). This is a more-than-twenty-five-fold reduction in the average effective dose in the CBCT cohort, which was 0.025 mSv (SD 0.01). There was a statistically significant difference between the two cohorts with *p* < 0.0001 (95% CI 0.60–0.64). This study demonstrates that intraoperative CBCT delivers a significantly lower effective dose to patients compared with postoperative CTFB. Where facilities exist, CBCT offers a safer and more efficient alternative, with future work needed to assess staff dose, wait times, and cost impacts.

## 1. Introduction

In recent years, the use of intraoperative computerised tomography (CT) for the surgical management of facial fractures has become more prevalent [1]. Intraoperative imaging allows for real-time visualisation of fracture reduction and fixation. This may become to be considered the gold standard of treatment for these fractures, much like the use of intraoperative fluoroscopy for the operative management of hand fractures, since its initial description by Coltman in 1948 [2].

In most facial fracture patterns, it is standard of care to obtain postoperative imaging of the facial bones following their surgical fixation [3,4]. This is often performed following the patient’s recovery from anaesthesia, necessitating clinical observation of the patient and admission to a hospital ward, and balancing the use of radiology resources with competing priorities. These factors may result in admission overnight or even for multiple days, prolonging hospital stay and patient care. The use of intraoperative imaging obviates the need for postoperative surgical scans [5,6], decreases the return to theatre rates [7], and allows for better appreciation of a complex operative landscape in almost real time.

Despite a slightly increased length of operation [8] and a higher intraoperative revision rate [9], the benefits of real-time intraoperative guidance in the fixation of facial fractures cannot be ignored. An important consideration in the use of intraoperative imaging is the exposure of both patients and surgical staff to radiation. Despite obviating the need for postoperative imaging, the consideration of patient (and staff) safety must remain the paramount priority in surgical care. The concern relating to increased radiation is of particular importance in patients with facial fractures undergoing surgical management, as they often require multiple imaging investigations throughout the course of their treatment.

Although not explored in facial fracture management, it is yet to be determined through the literature whether the use of intraoperative CT scanning confers a lower radiation dose (calculated as the effective dose, millisieverts, mSv) compared with a standard postoperative CT of facial bones (CTFB) using a conventional multidetector CT. This is important in minimising the radiation dose exposure to the patient. Intraoperative CT scanning has been shown in other surgical contexts to reduce patient effective dose [10,11], particularly when dose-saving protocols are utilised. Despite the decrease in radiation exposure to the patient, there is increased occupational exposure of radiation for staff in the operating theatre at the time of intraoperative CT use [12,13].

This study aims to examine the effective radiation doses in patients undergoing surgical management of their facial fractures with the aid of a fixed-arm CT. This study further aims to discuss methods seeking to minimise the radiation risk to the staff involved in patient care, and to demonstrate that the use of intraoperative imaging in facial fracture management can be a powerful tool in improving the accuracies and efficiencies of patient care.

## 2. Materials and Methods

### 2.1. Study Design

This retrospective cohort study was conducted at a tertiary teaching hospital. Inclusion criteria were adult patients who underwent surgical fixation of their facial fracture pattern undertaken by a single surgeon from July 2023 to November 2024. Exclusion criteria involved patients with these fracture patterns whose procedures were not undertaken in the hybrid surgical suite of our institution, or those who did not have intraoperative imaging performed. Institutional ethics committee approval was obtained (reference number QA24-117-108307).

### 2.2. The Hybrid Surgical Suite

The hybrid surgical suite in our institution is equipped with the Siemens Artis Pheno (Siemens Healthineers, Erlangen, Germany). This is a fixed-arm, cone-beam CT (CBCT) available for use at our institution in a hybrid operating suite built primarily for concurrent surgical and interventional radiology-based procedures. Usage of this suite and its equipment requires staff members in the hybrid operating suite to wear lead gown protection while use of the CBCT is underway. Operating the CBCT also requires trained radiographers familiar with the machine and its protocols.

### 2.3. Scanning Protocol

The protocol utilised by the fixed-arm CBCT scan was the “4sDCT Head Care”. This protocol follows 4 s acquisitions, utilises the Siemens CT reconstruction of Dyna Computed Tomography (DCT), on the Head Setting, at low dose. This protocol uses 70 kilovolts of energy, spins at a 200-degree rotation at 0.8 degrees per frame and takes 248 frames of images.

### 2.4. Data Collection

Data were collected on eligible patients including age, sex and date of surgery. Data on mechanism of injury or type of surgical fixation required were not collected as this was not the focus of the study. The effective dose of radiation was calculated by collecting dose area product (DAP) data for each of the intraoperative doses received by each of the patients. DAP is defined [14] as the product of the dose of radiation and the area irradiated, measured in Gy/cm^2^, and is used to assess the radiation exposure associated with imaging procedures. For use as a control, DAP data was also collected for a standard CTFB undertaken by 30 consecutive adult patients undergoing CTFB at our institution. The scan protocol used for these standard CTFBs was the standard CTFB protocol utilised by our institution.

### 2.5. Effective Dose Calculations

All CT dose simulations were performed using NCICT version 3.0 [15,16]. The required input parameters—scan range, tube voltage (kVp), patient sex, and volume CT dose index (CTDIvol)—were obtained from CT dose reports and patient records. Simulations were conducted using the 16 cm CTDI phantom to reflect standard head protocols. Tube current modulation was not included; a constant tube output was assumed for all scans. To ensure consistency in anatomical coverage for comparison, the simulated scan range was standardised from just above the frontal sinus to the menton.

**Figure 1:**
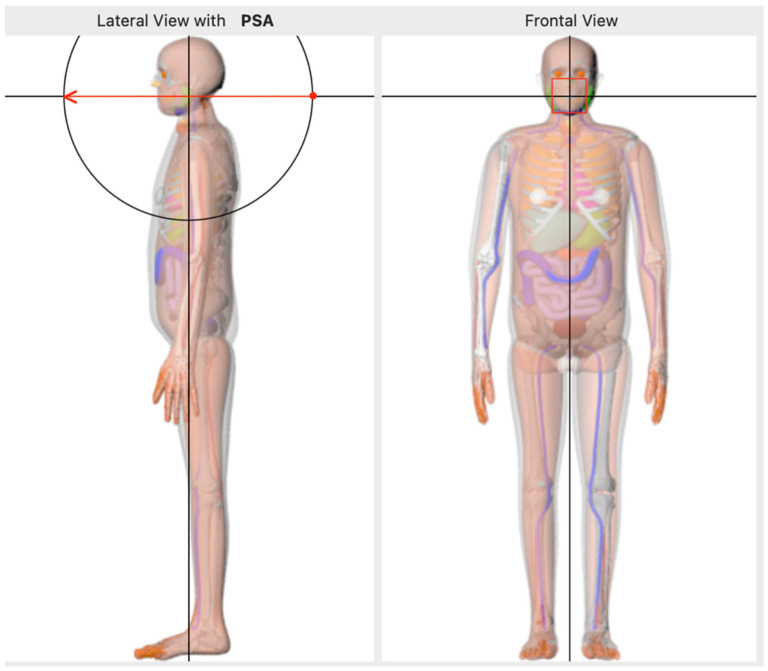
Isocentre positioning at head level shown in lateral and frontal views, with simulations performed over a 240° arc in 10° increments.

CBCT dose simulations were performed using NCIRF version 3.0. Input parameters were extracted from CBCT dose reports and included: patient sex, tube voltage (kVp), source-to-image distance (SID), field of view (FOV), and dose area product (DAP). The table thickness was fixed at 1 cm, and the number of histories was set to 10,000,000. The isocentre position was defined as shown in Figure 1. Simulations were conducted over a 240-degree arc in 10-degree increments, with the total DAP evenly distributed across all projections.

### 2.6. Data Analysis

Effective doses as calculated using the above simulations for both experimental (CBCT) and control (CTFB) cohorts were analysed for significance. Data analysis demonstrated normality of data and as such, unpaired t-testing on the effective dose data between the two cohorts was utilised. This was undertaken using GraphPad Prism 11 for MacOS (GraphPad Software, version 11, Boston, MA, USA).

## 3. Results

Within the study period, 48 patients underwent surgical fixation of their facial fractures. Due to incomplete or irretrievable data, the data of 24 patients were included in this study. Of these 24, the majority were male (17/24, 70.8%). The mean age of the population at the time of surgical fixation of their facial fracture was 49 years (range 20–94 years old, standard deviation 26.6 years). The intraoperative revision rate for our study was 12.5% (3/24).

**Figure 2:**
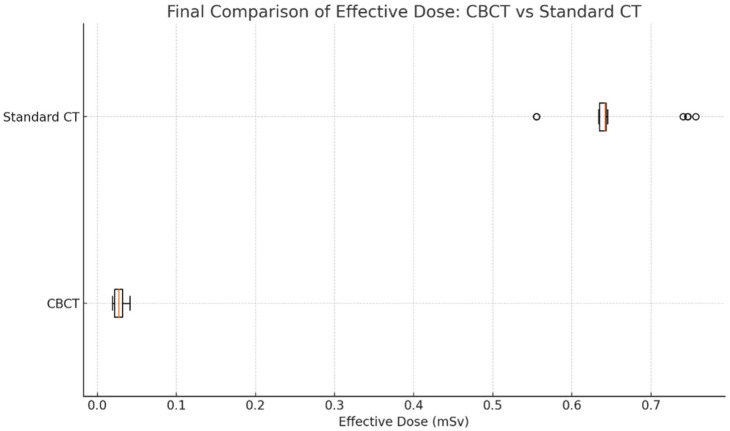
Boxplot comparison of effective dose between CBCT and conventional postoperative CTFB. CBCT demonstrated significantly lower effective doses (mean 0.025 mSv, SD 0.01) compared with CTFB (mean 0.64 mSv, SD 0.05), mean difference of 0.615 mSv, *p* < 0.0001 (95% CI 0.60–0.64).

The average dose of the effective doses calculated in the CTFB cohort was 0.64 mSv (standard deviation 0.05). Comparison yields a more-than-twenty-five-fold reduction in the average effective dose in the CBCT cohort, which was 0.025 mSv (SD 0.01). There was a statistically significant difference between the two cohorts with *p* < 0.0001 (95% CI 0.60–0.64). The results are further outlined in Figure 2 below.

## 4. Discussion

This study analyses the differences in effective radiation dose between the types of scans patients receive when their facial fractures have been managed surgically. We have demonstrated that a CT performed intraoperatively, using CBCT in the hybrid suite, confers a lower radiation risk to the patient compared with a standard postoperative CTFB. A key strength of this study is the direct comparison of effective radiation doses between a CBCT and a CTFB. Although it is well recorded in the literature that CBCT confers less effective radiation dose to the patient, to date, this study is the first to compare these two modalities directly in the context of surgically managed maxillofacial trauma. Our data demonstrates that the CBCT delivers 25 times (significantly) less radiation than CTFB. While this finding is supported by other dentomaxillofacial settings [17,18,19], our study is novel in that it is the first to outline this difference in the setting of acute bony maxillofacial trauma.

### 4.1. Benefits of CBCT

The reduction in the effective dose received by the patients when undergoing intraoperative CBCT is not the only benefit. Intraoperative imaging provides real-time feedback on the facial anatomy during surgery. This enables immediate assessment of surgical reduction and fixation so that corrections can be made intraoperatively [5], allowing for greater accuracy of reconstruction [20]. The intraoperative revision rate for fractures when utilising intraoperative scanning has been described in the literature as upwards of 20% [1,9,21]. Our study had an intraoperative revision rate below this at 12.5%, which may be reflective of the small sample size or the single surgeon and their experience at our institution. It has previously been suggested that suboptimal results are often accepted when further improvement of facial symmetry or the need to seek “better” fixation is weighted against the burden of a subsequent anaesthetic or surgery [22]. As such, any method by which the fixation and reduction of fractures might be improved, without these risks, is beneficial in patient care.

Prior to the use of the intraoperative CBCT, the gold standard of care at our institution (and many others) included a postoperative CTFB to determine fixation adequacy. As such, patients would therefore have to wait for such a scan to occur prior to being cleared for discharge, or to determine whether an admission is necessary to facilitate any further surgical correction of inadequate fixation and/or reduction. Although data on the time spent waiting for a CTFB postop was not collected, anecdotally, awaiting a scan can take upwards of 3 h depending on the workload of the medical imaging department. A prior study highlighted that the use of intraoperative CT added 14.5 min to their cases overall [8]; this timeframe is still much smaller than the time spent by patients awaiting a CTFB, even if anecdotal, as no CT scan will occur 14.5 min after it has been ordered.

Cost analysis has previously been performed when directly comparing intraoperative CT scans (i.e., CBCT) to routine postoperative CT (i.e., CTFB), demonstrating that it is overall less expensive to utilise the CBCT when taking into consideration revision rates, return to surgery, and theatre and hospital admission costs [23]. Although our study did not perform a fiscal analysis, it seems logical that CBCT would incur less of a financial burden when taking into consideration hospital admission time spent waiting for imaging, returning to theatre for potential revisions, or even overall hospital length of stay.

Intraoperative imaging has been used extensively in other specialties [24,25], including in maxillofacial surgery. However, the differing imaging modalities previously described rarely include a fixed-arm, cone-beam CT like the one we have utilised in our hybrid suite (e.g., mobile scans and mobile O-arms [21,26,27]). As may be anticipated, there has therefore been a learning curve in the use and optimisation of this modality in the setting of maxillofacial trauma.

### 4.2. Developing a Protocol

The wide range of CT doses observed in this study reflects the variability in clinical protocols, with some patients undergoing low-dose facial bone scans and others receiving higher-dose combined brain and facial bone imaging, which typically requires greater tube output to penetrate the skull. In addition, protocol choice (e.g., low-dose CTFB vs. combined brain and facial bone imaging) remains vitally dependent on patient presentation and injuries. Optimisation will always be considered on an individual case basis. However, this variability in CT protocols remains a source of bias and is a potential limitation in our study.

The first 5–8 cases of the cohort were the first cases undertaken in the hybrid suite at our institution. As such, a protocol had not yet been utilised and streamlining of the process occurred within these first few cases. This included collimating to the area of interest, limiting the area scanned, and increasing the arc of rotation of the scan to reduce images per spin without sacrificing the visualised gross anatomical landscape of the operative field in real time. Techniques such as these are well described in the literature, but overall reduce the radiation received by the patient, as well as the scattered radiation received by those surrounding the operative field, including the surgical and anaesthetic staff [28].

Our protocol includes staff exiting the operating theatre while imaging occurs. In our study, staff were initially required to wear lead aprons as a protective measure against radiation exposure. Despite its protective features, the use of lead aprons has been shown to increase back pain [29], and musculoskeletal injuries [30], posing occupational health and safety issues. Following the revision of our protocol, staff are now requested to leave the room when screening to the adjacent control room present within the hybrid suite, which has a window for observing the operating theatre. This is possible as the patient is under general anaesthesia and is also able to be monitored from this adjacent room. The need for staff to wear protective lead aprons is thus obviated, conferring the benefits of increasing comfort, reducing occupational health and safety risks, and eliminating the radiation exposure received by operating theatre staff.

### 4.3. Limitations

Despite the advantages of intraoperative CT scans that can aid in maximising patient outcomes and minimising radiation doses, not all hospitals are equipped with a surgical suite that has intraoperative scanning capacity. As such, a limitation of our study is the acknowledgement that the decreased effective radiation dose is only achievable in hospitals that can accommodate intraoperative scanning. However, our findings support the use of this when facilities are available and recommend that the standard of care be upheld at your institution regardless of intraoperative scanning capacity. 

Another limitation of our study is that 24 of the 48 eligible patients who had their facial fractures fixed were included, highlighting a 50% non-inclusion rate. This was due to irretrievable or incomplete dose and DAP data from the intraoperative scans themselves. This included information required to calculate the effective radiation dose; as such, lack of data for a particular patient meant that the patient could not be included in our study. Although it is unclear why the data was incomplete or irretrievable, it highlights the small patient cohort of our study and the need for a larger sample size in future work looking to mitigate a large exclusion rate. Furthermore, the lack of matched or paired comparison controls is another limitation in our study. Our study aimed to highlight the difference between the two populations and as such, a random cohort of 30 patients who underwent a CTFB with standard scanning protocol was used. However, we acknowledge this as a potential source of bias.

Data on the type of fracture was not gathered (e.g., fracture type or severity of injury). The severity of the injury and its resultant scan influence the effective radiation. We acknowledge this as a bias and a limitation in our study. Future studies could highlight the difference in the fractures fixed and stratify the radiation dose for more robust comparisons.

More future directions highlighted by this pilot study could include the analysis of hospital wait times for CTFB, or the analysis of received radiation by the surgical staff to further cement the notion that intraoperative CBCT is overall a safer approach for ensuring exemplary fracture fixation and patient care.

## 5. Conclusions

This study demonstrates that intraoperative CBCT delivers a significantly lower effective dose to patients compared with postoperative CTFB. Although variability in CTFB protocols contributes to a wide range of doses, optimisation should remain case-specific. Where facilities exist, CBCT offers a safer and more efficient alternative, with future work needed to assess staff dose, wait times, and cost impacts.

## Data Availability

The raw data supporting the conclusions of this article will be made available by the authors on request.

## Abbreviations

The following abbreviations are used in this manuscript:

CBCT	Cone-beam computed tomography
CT	Computed tomography
CTFB	Computed tomography–facial bones
DAP	Dose area product
FOV	Field of view
NCIRF	National Cancer Institute dosimetry system for Radiography and Fluoroscopy
SID	Source-to-image distance
